# Beyond quality of life: a cross sectional study on the mental health of patients with chronic kidney disease undergoing dialysis and their caregivers

**DOI:** 10.1186/s12955-017-0646-4

**Published:** 2017-04-17

**Authors:** Beatriz dos Santos Pereira, Neimar da Silva Fernandes, Nayara Pires de Melo, Renata Abrita, Fabiane Rossi dos Santos Grincenkov, Natália Maria da Silva Fernandes

**Affiliations:** 1 Psychology, Federal University of Juiz de Fora (UFJF) – Interdisciplinary Nuclei of Nephrology Studies and Researches (NIEPEN), Juiz de Fora, Brazil; 2 Exact Sciences, Federal University of Juiz de Fora (UFJF), Juiz de Fora, Brazil; 3 Social Services, Federal University of Juiz de Fora (UFJF), Juiz de Fora, Brazil; 4 Psychology, Higher Education Center of Juiz de Fora (CES), Juiz de Fora, Brazil; 5 Psychology University, Federal University of Juiz de Fora (UFJF), Juiz de Fora, Brazil; 6 Medical Clinic Department, Federal University of Juiz de Fora (UFJF), Rua Jamil Altaff, 132, Vale do Ipê, Juiz de Fora, Minas Gerais Brazil CEP- 36035 380

## Abstract

**Background:**

Patients with terminal chronic kidney disease (CKDT) requiring renal replacement therapies (RRT) undergo important changes in living habits and frequently need caregiving. These patients and their caregivers are risk groups for the development of physical and psychological symptoms. This study aimed to evaluate the prevalence of anxiety, depression, stress, fatigue, social support, and quality of life in patients with CKD and their caregivers.

**Method:**

This cross sectional study was conducted with 21 patients and their caregivers, from January to September 2015. We included patients aged over 18 years, with at least 6 months on dialysis treatment, and caregivers who were family members. The participants’ social, demographic, clinical, laboratory, and psychological variables were evaluated. A descriptive analysis and an examination of the association between patients and caregivers were performed.

**Results:**

Among patients, we observed that 38.1% had symptoms that indicated anxiety and depression. The average score for practical social support was 3.15 ± 0.769 and that for emotional social support was 3.16 ± 0.79. As for fatigue, 14.3% of patients reported being ‘extremely tired’ and 14.3% reported that they engaged in all the activities they usually performed before the illness. Further, 57.1% presented stress, and of these, 66.7% were at the resistance stage, with predominance of psychological symptoms in 60.0%. The quality of life domain in terms of functional capacity (FC) presented a correlation with haemoglobin level (*r* = 0.581, *p* = 0.006) and non-anaemic patients presented better FC. Among caregivers, we observed symptoms that indicated anxiety and depression in 33.3% of the sample. Caregivers exhibited an average score of 2.88 ± 0.77 for practical social support and 3.0 ± 0.72 for emotional social support. Further, 14.3% reported being ‘extremely tired’ and 28.8% reported that they engaged in all activities that they usually performed before the patient’s illness. When comparing the two groups (patients vs. caregivers), we observed that they presented similar results for the presence of anxiety, depression, and fatigue. Caregivers received less social support than patients did. Both groups presented similar predominance of stress levels; however, patients presented more predominance of psychological symptoms. With reference to quality of life, patients and caregivers presented similar results on the social aspects, vitality, mental health, and mental domains.

**Conclusion:**

The mental health characteristics of patients and caregivers were similar, and within the context of dialysis for renal disease, both must undergo specific interventions.

## Background

The demographic transition process, characterised by a decrease in mortality and fecundity rates, and population ageing, leads to changes in the country’s morbidity standards, with a significant increase in the predominance of chronic non-communicable diseases [1]. Within this context, chronic kidney disease (CKD) appears as a public health problem, because of its predominance, evolution, and financial cost [2].

As a therapeutic resource, CKD relies on conservative treatment (CT); however, when the patient reaches a very low glomerular filtration rate (GFR) (that less than 10 ml/min/1.73 m^2^), there is a need to start renal replacement therapy (RRT) [3, 4]. RRTs constitute the provision of a support system for kidney functions and require important changes in lifestyle that generate the need for patients to adjust their social life, which are also related to the physical limitations resulting from this process [4]. These situations compromises several aspects of the patient’s life, such as physical, social, family, and financial, requiring them to adapt to the intense changes caused by the diagnosis and progress of the disease [2]. It renders the patient now dependent on multiple forms of care and on caregivers [5, 6].

Within this context, caregivers, mainly family members, represent a risk group for the development of psychological symptoms and several chronic diseases. Therefore, it is essential to evaluate the progress of the experience of becoming sick. However, our reality evidences the lack of and need for studies and interventions that focus on the impact of the disease on patients with CKD and their support group/family [7].

Few studies have addressed the patient–caregiver dyad. For instance, Fan et al. [8] demonstrated that, at the beginning of treatment, patients and caregivers presented similarities in their mental health, with the development and improvement of social function. However, as the disease progressed, caregivers of highly dependent chronic kidney patients (with daily dialysis) presented worsening of their mental health when compared to those of less dependent patients.

Among the psychological consequences found in patients with CKD and their relatives, depression was identified as the most common disorder. According to the studies by Rioux et al. [9] and Arechabala et al. [10], caregivers and patients presented similar mental health conditions, in that both met the criteria for depression as a self-perceived condition, and the symptoms worsened with time.

This situation negatively influences the quality of life of the subject and his/her caregivers, as well as decreases adherence to treatment and increases the rate of clinical complications and mortality [11–13].

The purpose of this study was to evaluate the prevalence of anxiety, stress, depression, and fatigue symptoms, and the levels of quality of life and perceived social support in patients with CKD and their caregivers. Additionally, it aimed to examine the relationship of these variables with patient’s clinical characteristics.

## Method

### Study design and period

This cross sectional study on family caregivers and patients with CKD undergoing RRT [haemodialysis (HD)/peritoneal dialysis (PD)] was conducted at the hospital of the Federal University of Juiz de Fora (UFJF), from January to September 2015.

This study followed the standards recommended in the Declaration of Helsinki and was approved by the Health Ethics and Research Committee (36345514.1.0000.5139 under number: 081979/2014).

### Sample

Convenience sampling was employed, and 64 participants were approached, of which 30 were patients and 34 were caregivers. Of those, 5 patients did not have caregivers, and, in spite of our initial plan to create a group of patients with no caregivers, this number was very small. Therefore, these patients were excluded from the analysis. Four patients with caregivers who refused to be a part of the survey, and 13 caregivers with patients who refused to participate and did not sign the Free and Informed Consent Terms were excluded. Finally, data from 21 pairs of patients and caregivers were analysed.

Caregivers were considered as the person indicated by patients as ‘responsible for taking care of their health at home’.

Inclusion criteria: CKD patients and family caregivers who were older than 18 years of age, from both genders, had been undergoing RRT for at least six months, and who accepted to participate in the survey and signed the Free and Informed Consent Terms were selected for the present study. Exclusion criteria: patients who presented difficulty in understanding the questionnaires or had records of serious cognitive deficits in their medical charts, and those who refused to sign the Free and Informed Consent Terms, were excluded from the sample.

### Procedures

In patients on HD, the survey was conducted during the sessions. For patients treated through PD, the survey was conducted during their medical appointments. In case of caregivers, the interview was performed at the waiting room of the medical clinic, when they visited with their patient.

### Variables

The following social and demographic data of patients and caregivers were evaluated: age, gender, ethnicity, level of education, and marital status (according to the Brazilian Institute of Statistical and Geography [IBGE]). Additionally, the following psychological variables were collected using specific instruments: anxiety, depression, perceived social support, fatigue, stress level, and quality of life. For patients, the following additional clinical data were also collected: CKD aetiology, comorbidities, and laboratorial data; haemoglobin (Hb) and haematocrit (HTC) levels; saturation index for transferrin and ferritin; calcium, phosphate, alkaline phosphatase, and parathyroid hormone (PTH) levels; and creatinine, urea, and Kt/V levels.

Anxiety and depression were measured using the Hospital Anxiety and Depression Scale (HADS) [14], level of perceived social support through the Scale of Perceived Social Support (SPSS) [15], Fatigue levels through the Fatigue Pictogram [16], stress level and stage through the Lipp’s Stress Symptoms Inventory (LSSI) [17], and quality of life through the SF-36 Quality of Life Scale [18] (Table 1).

**Table 1:** Research instruments used in the study

Instrument	Characteristics	Recommended method of application
Social and demographic questionnaire	This semi-structured interview constituted of questions about the participant’s social and demographic data, and was prepared by the research team.	Non-private instrument from the psychologist
Hospital Anxiety and Depression Scale (HADS - AD)	It evaluates the presence of anxiety and depression symptoms on a Likert-type scale with 14 items [7 for HADS-Anxiety (HADS-A) and 7 for HADS-Depression (HADS-D)]. Each item is scored with values from zero to three, constituting a maximum score of 21 points for each scale. The cut-off point for the presence of symptoms is a score of ≥ 9 value.	Non-private instrument from the psychologist
Scale of Perceived Social Support (SPSS)	This instrument evaluates the social support perceived by the individual. It comprises 29 items with answers rated from 1 to 4 to evaluate two dimensions of perceived social support, practical and emotional.	Non-private instrument from the psychologist
Fatigue Pictogram	This illustrated instrument evaluates fatigue. It presents two sets of figures that evaluate the intensity and impact of fatigue in regular activities. Figures are presented on an ordinal scale constituted by two questions with five graduated and captioned illustrations that evaluate the intensity and impact of fatigue. This tool does not have a cut-off point for the diagnosis or classification of intensity of fatigue.	Non-private instrument from the psychologist
Lipp's Stress Symptoms Inventory (LSSI)	This self-report instrument identifies the presence of stress symptoms, type of existing symptoms (somatic or psychological predominance), and stage of stress (alarm, resistance, near-exhaustion, and exhaustion).	Private instrument from the psychologist
Quality of Life Questionnaire (SF-36)	This summarised version of the Medical Outcomes Trust questionnaire evaluates several quality of life domains by attributing scores (0–100), with scores closer to zero indicating worse quality of life and those closer to one hundred indicating better quality of life.	Non-private instrument from the psychologist

### Statistical analysis

Qualitative data (gender, fatigue intensity, etc.) were described using frequencies and percentages; quantitative variables (haemoglobin level, age, etc.) were evaluated using mean, standard deviation, minimum, and maximum scores. To correlate the obtained variables for patients and their respective caregivers, the Pearson’s and Spearman’s correlation coefficients were used and were selected according to the type of variables. To analyse the difference in the central trends of the patient/caregiver pair along the study variables, the Wilcoxon’s non-parametric test was used. The significance level was set at *p* ≤ 0.05. The statistical software SPSS-17©, Chicago, Illinois was used for all analyses.

## Results

### Patients

In this study, out of the 21 patients evaluated, 33.3% were undergoing PD and 66.6% were undergoing HD. Their age ranged from 29 to 80 years, with an average of 58.9 ± 14.4 years. Further, 57.1% were female, 47.6% were black, and treatment time varied from seven months to 18 years, with an average of 4.8 ± 4.7 years. Additionally, 9.2% of patients were illiterate and 90.5% of the patients were not working during the study period. Out of the ones who worked, 50% had full time jobs. All patients presented high blood pressure, while 33.3% presented diabetes mellitus. The most prevalent cause of CKD was diabetic nephropathy (33.3%) and hypertensive nephrosclerosis (23.8%) (Table 2).

**Table 2:** Social and demographic data of patients and caregivers

	Patients (*n* = 21)	Caregivers (*n* = 21)
Age in years (mean, dp)	58,9 ± 14,4	47,86 ± 15,21
Female (%)	12 (57,1%)	16 (76,2%)
Ethnicity (%)	Black 10 (47,6%)	Black 6 (33,3%)
	White 8 (38,1%)	White 8 (44,4%)
	Mixed Race 3 (14,3%)	Mixed Race 4 (22,2%)
Marital Status	Married 47.6% (10)	Married 61.9% (13)
Level of education	9.2% (2) of patients were illiterate Higher education was evidenced for 9.5% (2)	85.7% (18) had graduated from High School and only 14.3% (3) graduated from University
Place of origin	28.6% (6) came from Juiz de Fora	42.9% (9) came from Juiz de Fora
Work	90.5% (19) of patients did not work	66.7% (14) had some form of occupational activity
Type of Treatment	14 (66.6%) undergoing HD 7 (33.3%) undergoing DP	–
Treatment Time in years	4,8 ± 4,7	–

**Fig. 1:**
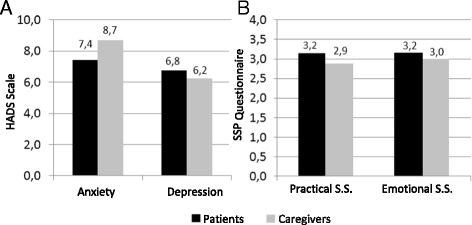
**a** -Anxiety and Depression in Patients and Caregivers. **b**- Social Support in Patients and Caregivers

An assessment of the presence of anxiety and depression symptoms showed that 38.1% presented symptoms indicating anxiety and 38.1% had symptoms indicating depression (Fig. 1).

On evaluating the perceived social support among the 21 patients, we observed that, for practical support, the minimum score was 1.11 while the maximum score was 3.89, with an average of 3.15 ± 0.769. For emotional support, the minimum score was 1.30 and maximum was 4.00, with an average of 3.16 ± 0.79 (Fig. 1).

**Fig. 2:**
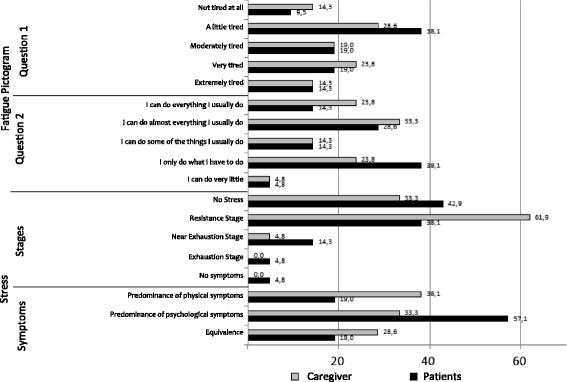
Fatigue and Stress in Patients and Caregivers

When patients were questioned ‘how tired have you felt over this last week?’, 14.3% answered ‘extremely tired’ and 9.5% stated that they were ‘not tired at all’. However, when asked if this feeling of tiredness prevented them from doing what they wished, 4.8% reported that ‘I can do very little’ and 14.3% said ‘I can do everything I normally do’ (Fig. 2).

An analysis of the presence of stress symptoms and their level, stage, and symptom type predominance (physical or psychological) showed that, out of 21 patients, 57.1% presented stress. Of those, 66.7% were at the resistance stage, 25.0% were at the near-exhaustion stage, and 8.3% were at the exhaustion stage. Of the ones diagnosed with stress, 20.0% presented predominance of physical symptoms, 60.0% presented predominance of psychological symptoms, and 20.0% presented both symptoms in equal proportions (Fig. 2).

Based on the amplitude of the laboratory results, we selected a group of variables that can summarise the patient’s health conditions related to this study. Thus, we used the Hb, Kt/V, and urea levels for further analyses. Several parameters are used to evaluate the adequacy of dialysis. The ones chosen in this study are the most relevant ones. The Kt/V level relates to an adequacy rate based on urea depuration. The patients on HD in this study underwent a monthly evaluation. However, there are no specific guidelines for the frequency of evaluation of patients on DP. In our case, it was annual, and Kt/V level was not among the variables analysed for patients undergoing RTT in this mode [19]. Therefore, the correlation between laboratory and psychological variables, and treatment time was evaluated (Table 3).

**Table 3:** Correlation between laboratory and psychological variables and treatment time for patients

	Urea			Hemoglobin			Kt/v		
	r	*p*-value	n	r	*p*-value	n	R	*p*-value	n
Functional Capacity^a^	0,230	0,317	21	0,581	0,006	21	−0,432	0,123	14
Limitation by physical aspects^a^	0,068	0,771	21	0,169	0,463	21	−0,032	0,914	14
Pain^a^	−0,019	0,936	21	0,194	0,399	21	0,103	0,727	14
General health conditions^a^	−0,005	0,982	21	−0,288	0,206	21	−0,038	0,899	14
Vitality^a^	−0,121	0,602	21	−0,009	0,970	21	−0,190	0,515	14
Social aspects^a^	0,237	0,302	21	0,173	0,454	21	−0,195	0,503	14
Limitation by emotional aspects^a^	0,035	0,879	21	0,184	0,425	21	0,015	0,961	14
Mental health^a^	−0,105	0,650	21	0,185	0,423	21	−0,487	0,077	14
Physical Domain^a^	0,163	0,481	21	0,258	0,259	21	−0,068	0,817	14
Mental Domain^a^	−0,055	0,812	21	0,077	0,742	21	−0,276	0,339	14
Hospital Anxiety Scale^a^	−0,078	0,737	21	−0,003	0,991	21	0,166	0,571	14
Hospital Depression Scale^a^	−0,032	0,889	21	−0,087	0,707	21	0,288	0,318	14
Practical Social Support (patient)	0,321	0,156	21	−0,149	0,519	21	−0,327	0,253	14
Emotional Social Support (patient)^a^	0,105	0,650	21	−0,214	0,351	21	−0,222	0,445	14
“How tired have how tired have you felt over this last week?” (Question 1 on the Fatigue pictogram)^b^	−0,179	0,437	21	0,164	0,576	14	−0,028	0,903	21
“How much does the feeling of tiredness prevent you from doing what you want?” (Question 2 on the Fatigue pictogram)^b^	0,136	0,558	21	0,175	0,549	14	0,231	0,313	21
Stress Stages (According to Lipp)^b^	0,197	0,392	21	0,099	0,669	21	0,153	0,601	14

^a^Using the Spearman Correlation Coefficient

^b^Using the Pearson Correlation Coefficient

It has been observed that Hb, Kt/V and urea levels did not have a significant correlation with age and with the two questions related to evaluation of fatigue. Further, there were no significant correlations between laboratory exams and both dimensions of perceived social support. Stress evaluation, i.e., presence and predominance of either physical or psychological symptoms, and their stages, were also not correlated to laboratory variables. Similarly, the laboratory variables did not have a significant correlation with the presence of anxiety and depression symptoms (Table 3).

On analysing the domains of quality of life, we observed a significant correlation of functional capacity (FC) with Hb level through the Pearson’s correlation coefficient (*r* = 0.581, *p* = 0.006). This indicated that, the better the level of Hb was, the better was the patient’s FC. For illustration purposes, we observed that anaemic patients (8 patients) presented average FC scores of 40.00 ± 22.20 and non-anaemic patients (13 patients) had an average score of 61.153 ± 26.469 (*p* = 0.075). The same domain did not present a statistically significant result when its correlation with Kt/V (*r* = −0.432, *p* = 0.123) and urea (*r* = 0.230, *p* = 0.317) levels was examined (Table 4).

**Table 4:** Correlation between quality of life in patients and caregivers

	Patient		Caregiver				Correlations	
Variables	Average	Standard deviation	Average	Standard deviation	Z	*p*-value	*r*	*p*-value
Functional Capacity	53,09	26,52	74,52	26,64	−2,61	0,014	0,191	0,406
Pain	48,66	30,44	56,95	24,93	−0,96	0,344	−0,223	0,331
General health conditions	51,00	26,85	63,47	26,75	−1,51	0,179	−0,084	0,717
Vitality	58,81	28,37	44,52	27,29	1,66	0,198	−0,312	0,168
Social aspects	60,71	32,42	63,69	32,33	−0,30	0,913	0,360	0,109
Mental health	66,48	25,19	57,33	24,11	1,20	0,321	−0,079	0,732
Physical aspects	33,33	36,51	64,28	38,38	−2,68	0,006	0,334	0,138
Emotional aspects	41,26	40,69	55,55	45,13	−1,08	0,103	0,625	0,002
Physical Domain	36,36	8,96	46,29	10,29	−3,33	0,005	−0,021	0,929
Mental Domain	45,65	12,07	40,09	15,01	1,32	0,170	0,176	0,447
Anxiety	7,42	5,35	8,71	3,84	−0,928	0,37	0,077	0,741
Depression	6,76	4,79	6,23	4,41	0,419	0,71	0,227	0,322
Practical Social Support	3,15	0,76	2,88	0,77	1,309	0,27	0,278	0,222
Emotional Social Support	3,16	0,79	3,00	0,72	0,993	0,50	0,548	0,010

With reference to the SF-36 domains, only ‘limitation from emotional aspects’ (*r* = 0.472; *p* = 0.031) and ‘great mental domain’ (*r* = 0.370; *p* = 0.09) presented significant correlations. Anxiety, depression, social support, and stress were not related. Further, no significant relationship was observed between treatment time and fatigue (Table 4).

The most common type of therapy was HD (66.6%), and there was no difference between HD and PD, probably owing to the size of the sample.

### Caregivers

The study sample included 21 caregivers with the following social and demographic characteristics: age ranged between 26 and 76 years, with an average of 47.86 ± 15.21 years; 76.2% were female; majority of them were married, 61.9%; and 44.4% were white. Further, 85.7% had graduated from high school, and 66.7% were engaged in some sort of occupational activity at the time of the present study (Table 2).

Out of the 21 caregivers, 33.3% presented symptoms that indicated anxiety and 33.3% had symptoms that indicated depression (Fig. 1).

The minimum score on perceived practical support was 1.47 and the maximum was 4.00, with an average of 2.88 ± 0.77. As for perceived emotional support, the minimum value was 1.60 and maximum was 4.00, with an average of 3.00 ± 0.72 (Fig. 1).

As for how tired the caregivers were over the last week, approximately 14.3% reported being ‘extremely tired’ and 14.3% reported being ‘not tired at all’. When asked about the extent to which their tiredness had limited their activities, 4.8% stated ‘I can do very little’ and 23.8% said, ‘I can do everything I normally do’ (Fig. 2).

Regarding the presence of stress symptoms, out of the 21 evaluated caregivers, 66.7% presented stress symptoms. Of those, 92.9% were at the resistance stage and 7.1% were at the near-exhaustion stage, while none of the participants was at the alarm or exhaustion stages. Out of the ones diagnosed with stress, 38.1% presented predominance of physical symptoms, 33.3% presented predominance of psychological symptoms, and 28.6% presented both symptoms in equal proportions (Fig. 2).

**Fig. 3:**
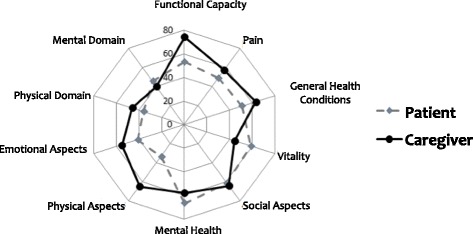
Quality of Life in Patients and Caregivers

Scores pertaining to the quality of life, as assessed using the SF-36, have been presented in Fig. 3.

### Correlation between patient and caregiver variables

There was no significant correlation between the patients’ and caregivers’ age (*r* = 0.269, *p* = 0.238).

When evaluating the presence of anxiety and depression symptoms, we observed that patients and caregivers presented very similar averages for both symptoms (Fig. 1) and there was no significant correlation (Pearson’s correlation coefficient) between the patients’ and caregivers’ results. In the analysis to examine differences in anxiety and depression using the Wilcoxon test, no significant differences were observed for anxiety, with patients presenting an average of 7.43 ± 5.35 and caregivers an average of 8.71 ± 3.85 (z = −0.802, *p* = 0.423). Similar findings were observed for depression, with patients presenting an average of 6.76 ± 4.79 and caregivers an average of 6.24 ± 4.41 (z = −0.423, *p* = 0.672).

On comparing the perceived social support between patients and caregivers, again, very similar values were observed. However, even with close values, we observed that caregivers presented smaller averages than patients did, indicating that caregivers received less social support, either practical or emotional (Fig. 1). There was no significant correlation between the practical social support scores of patients and caregivers; however, there was significant correlation between their emotional social support scores (*r* = 0.548, *p* = 0.010).

In another statistical approach, the perceived social support was examined with reference to practical and emotional aspects; however, no statistical significant findings were observed. For practical social support, patients presented an average of 3.15 ± 0.77 and caregivers an average of 2.89 ± 0.78 (z = −1.165, *p* = 0.244), as for emotional social support, patients presented an average of 3.17 ± 0.80 and caregivers an average of 3.01 ± 0.72 (z = −1.121, *p* = 0.262).

When evaluating fatigue levels and their interference in daily activities, when asked ‘How tired have you felt over this last week?’, patients and caregivers presented similar percentages distribution of fatigue levels (*p* = 0.89). The same was observed with reference to the patients’ and caregivers’ responses to ‘To what extent does the feeling of tiredness prevent you from doing what you want?’ (*p* = 0.30). There were no significant correlations between patients’ and caregivers’ responses for either of these questions (*r* = −0.215, *p* = 0.349; *r* = −0.045, *p* = 0.847, respectively).

Excluding the cases of absence of stress, on examining the predominance of physical/psychological symptoms/both in the patients and their caregivers, we observed that patients tended to present more psychological stress symptoms (75%, 16) when compared to caregivers (46.7%, 15). However, this result did not reach statistical significance (*p* = 0.149). However, there were no differences between patients and caregivers in terms of the presence of stress (*p* = 0.751) (Fig. 2).

Regarding stress stages, both groups presented similar results (*p* = 0.967). There was no difference between the groups for the incidence of physical symptoms (*p* = 0.640). However, patients tended to be more psychologically stressed than caregivers did, though the observed difference was not statistically significant (*p* = 0.071). For predominance of physical or psychological symptoms, including patients with no stress symptoms and cases that showed both symptoms equally, no significant difference was observed (*p* = 0.220).

There was no significant correlation between the presence and stages of stress (*r* = 0.070, *p* = 0.764), i.e., the intensity of patient stress was not related to the intensity of caregiver stress. On evaluation of the predominance of physical or psychological symptoms, the Chi-square test presented no relationship between the categories (*X* ^2^ = 2.213, *p* = 0.137) indicating that the predominance of a certain symptom in patients is not related to any physical/psychological characteristic of the caregiver.

With reference to the SF-36 scores, we observed statistically significant differences in that patients presented less FC in relation to caregivers, with patients achieving an average of 53.092 ± 26.527 and caregivers an average of 74.523 ± 26.641 (*z* = −2.453, *p* = 0.014). Patients exhibited more limitations due to physical aspects, and they presented an average of 33.33 ± 36.51, while caregivers presented an average of 64.28 ± 38.38 (*z* = −2.72, *p* = 0.006). As for the ‘physical’ domain, we observed that patients presented lower scores, with an average of 36.36 ± 8.96, while caregivers obtained an average of 46.29 ± 10.29 (*z* = −2.833, *p* = 0.005) (Fig. 3).

We observed that, for the physical domain, caregivers presented values that indicated higher quality of life pertaining to this aspect (Fig. 3).

Other clinically relevant findings were related to the sub-domains of vitality, social aspects, mental health, and the larger mental domain, with patients and caregivers presenting similar scores. In these categories, we also observed a tendency of caregivers to obtain lower scores, indicating comparatively worse quality of life in these aspects as compared to patients (Fig. 3).

Further, there was significant correlation in the scores on limitation by emotional aspects between patients and caregivers (*r* = 0.625, *p* = 0.002).

Even though some results did not reach statistical significance, the following findings are notable: in the ‘general health conditions’ domain, we observed an average of 51.00 ± 26.85 for patients and 63.47 ± 26.75 for caregivers, which demonstrates that patients presented lower scores (*z* = −1.345, *p* = 0.179), and for vitality, patients presented an average of 58.81 ± 28.37 and caregivers an average of 44.52 ± 27.29, which demonstrates that caregivers presented lower scores (*z* = −1.345, *p* = 0.179). Further, for limitation by emotional aspects, patients presented an average of 41.27 ± 40.69 and caregivers an average of 55.55 ± 45.13, where lower patient scores are evident (*z* = −1.628, *p* = 0.103), and for the mental domain, patients presented an average of 45.66 ± 12.07 and caregivers presented an average of 40.09 ± 15.10, which demonstrates that caregivers had lower scores on the mental domain, and lower quality of life in this aspect (*z* = 1.373, *p* = 0.170). Under the domains of pain (*z* = −0.947, *p* = 0.344), social aspects (z = −0.110, *p* = 0.913) and mental health (z = −0.992, *p* = 0.321) there were no significant differences (Fig. 3).

## Discussion

In this study, we observed that the prevalence of anxiety, depression, perceived social support, fatigue, stress symptoms, and levels of quality of life in patients with CKD and their caregivers was similar, and some variables presented worse scores in caregivers. Additionally, we evaluated the relationship of psychological variables between patients and caregivers, and found that some of them were related.

A recent study by Urquhart-Secord R et al., with 83 patients undergoing HD and their caregivers in Australia and Canada, evaluated 68 questions on outcomes in dialysis. They considered mortality, anxiety, and depression as the most relevant variables, as do most studies performed with this population [20].

In our study, the prevalence of anxiety and depression was similar to that reported in the literature. For instance, Arechabala et al. evaluated the presence of depression symptoms and their correlation to perceived social support and fatigue level with 162 pairs of patients and caregivers. It was observed that both groups presented similar levels of depression symptoms [10].

Social support, both practical and emotional, is vital for chronically ill patients and their caregivers. Our results showed that caregivers presented lower averages than patients did, even though there was no statistical significance. The correlation of emotional support between the two groups shows that patients who have more emotional social support tend to be cared for by caregivers who also have more social support in this aspect. This data reinforces the notion that other psychological variables, such as anxiety and depression, must be observed in caregivers to promote their mental health and their ability to provide support to patients. This result corroborates with those of studies conducted by Schwartz et al., which evidence the importance of the development of a support network to face CKD. It is recommended that this network constitute other family members, neighbours, and friends, and that it should incorporate the influence of spirituality on each individual, and the bond with healthcare professionals, to promote adhesion to treatment and to support patients to fight the disease [21].

Regarding fatigue, our study obtained similar results for patients and caregivers in relation to the level of fatigue and interference of fatigue in daily activities. In a study on the presence of fatigue in patients with kidney disease and their caregivers, Schneider highlighted that fatigue can be an important early symptom that indicates decrease in caregiver quality of life. Additionally, the physical and psychological conditions of this group, which are often neglected, may be important for the adjustment and recovery of patients with CKD [22].

When evaluating the stress presented by patients and caregivers in our study, we found similar results with reference to the presence of stress symptoms and stages. Patients also had the tendency to present more psychological symptoms than caregivers did. In a systematic review, in 2014, Garcia-Llana evaluated 38 studies on chronic diseases and found that only two of them had evaluated stress. These studies evaluated the impact of stress on quality of life, and both studies concluded that stress, mental, and physical health, evaluated together with quality of life, are indirectly related [23].

Quality of life in caregivers of chronic patients can be changed as a result of the caring process and also by the worsening of the disease. Pinto et al. evaluated quality of life in caregivers of patients with Alzheimer’s, using information from 118 participants. It was observed that the most compromised scores on the SF-36 were related to vitality, and physical and emotional aspects. Worse quality of life in caregivers was related to worse FC in patients, and it was found to have a negative impact on the care provided [24]. Similarly, in our study, when comparing patients and caregivers, patients presented lower scores on the physical domain, and for pain, the results were similar. Caregivers presented even lower levels of vitality and mental health. Results also demonstrated that patients who are more emotionally limited tend to be cared for by caregivers who are also more limited in this aspect.

Shiue and Sand [25] suggest that proper training and support for caregivers is necessary to decrease care overload. To demonstrate this, a population-based study was performed to evaluate quality of life in caregivers with and without previous chronic diseases. It was observed that caregivers with current chronic diseases (1.562) presented physical health limitations, body pains, and emotional problems, and they were less active. Caregivers with no chronic diseases (1.151) presented similar results [25]. In our study, caregivers presented lower quality of life in the domains of vitality and mental health.

Caregivers of patients undergoing dialysis also experience adverse effects to their quality of life, and therefore, interventions for educational, social, and psychological support are essential to promote their ability to handle adversities generated by this context, as highlighted by Celik et al. When comparing quality of life of 142 pairs of patients undergoing dialysis and their caregivers, and their symptoms of anxiety and depression, and quality of sleep, it was observed that both groups presented similar results for the physical and mental components of quality of life, and caregivers presented having more problems related to sleep [26].

Owing to the importance of family relationships to promote/generate risk health behaviours and the response of the patient to the disease, the biopsychosocial support model, that is widely used currently, intends to attend to and comprehensively intervene with individuals and their social environment, especially their family unit. These interventions intend to promote strategies to establish healthier and more organized families and communities to prevent the worsening of health and to help patients face adversities [27].

Our study has some limitations, information about the duration for which the caregivers had been playing this role was not obtained.

We concluded that patients and caregivers presented similar mental health levels and, interventions that consider both groups together and individually must be implemented.

## Conclusions

Support groups are well recommended for this population because such groups would help us cater to both patients and caregivers simultaneously, to help them adapt more easily to the RRT routine. Group treatment, in this situation, allows for greater acceptance and identification among the members, promoting benefits to physical and mental health. Additionally, studies using this approach are recommended.

## Abbreviations

CKD: Chronic kidney disease
CKDT: Terminal chronic kidney disease
CT: Conservative treatment
GFR: Glomerular filtration rate
HADS-AD: Hospital anxiety and depression scale
Hb: Haemoglobin
HTC: Haematocrit
IBGE: Brazilian Institute of Statistical and Geography
Kt/V: This variable relates to an adequacy rate based on urea depuration
PTH: Parathyroid hormone
LSSI: Lipp’s stress symptoms inventory
RRT: Renal replacement therapies
Sf-36: Quality of life questionnaire
SPSS: Scale of perceived social support
